# ALT (allogeneic limbal transplantation): a new surgical technique for limbal stem cell deficiency

**DOI:** 10.1007/s10792-022-02373-8

**Published:** 2022-08-19

**Authors:** Anja Viestenz, Christiane Kesper, Thomas Hammer, Joana Heinzelmann, Sabine Foja, Arne Viestenz

**Affiliations:** Department of Ophthalmology, University Hospital Halle (Saale), Martin-Luther-University Halle-Wittenberg, Ernst-Grube Straße 40, 06120 Halle, Germany

**Keywords:** Corneal burn, Limbal stem cell deficiency, Limbal stem cells, Allogeneic limbal transplantation, Penetrating keratoplasty

## Abstract

**Purpose:**

Limbal stem cell deficiency (LSCD) is a rare but extremely relevant disease of the eye. LSCD patients often require a variety of surgical procedures, including keratoplasty in some cases. However, the outcome of these surgeries, including opacification and revascularization, is often frustrating due to LSCD relapse.

**Methods:**

We developed a new surgical technique for the treatment of LSCD in which partial allogenic limbal transplantation (ALT) is carried out as part of penetrating keratoplasty (PK). After the PK, 1–8 slices from the limbal tissue of the donor graft are prepared and placed under the double running sutures attaching the corneal graft. This procedure was performed on 14 patients with LSCD, caused by severe ocular burn in 5 cases and by infection in 9. Between one and eight limbal transplants were used depending on the extension of the LSCD.

**Results:**

All 14 patients showed stable or increased visual acuity after the ALT surgery compared to their preoperative visual acuity. All of the grafts were integrated into the superficial corneal layers without progression of corneal vascularization beyond the limbal grafts. The median follow-up period was 12 months on average.

**Conclusion:**

The ALT method seems to be a promising surgical procedure for the treatment of patients with LSCD. It can be properly carried out in the context of keratoplasty and does not require a separate donor tissue. The ALT grafts may offer the possibility of constructing a new limbal region, resulting in stable or even increased visual acuity and the absence of corneal vascularization.

## Introduction

Limbal stem cell deficiency (LSCD) is a ocular disease caused by damage to the limbal stem cells. These stem cells are located at the limbus, and from their stem cell niche they are able to proliferate and differentiate, leading to continuous renewal of the corneal epithelium. Furthermore, they build a barrier to prevent the conjunctival epithelial cells from overgrowing the cornea, which is important for corneal transparency. Damage to this limbal region, for example due to chemical burn, often leads to LSCD with a massive loss of visual acuity, conjunctivalization of the cornea, and multiple corneal surface defects. In many cases, the patients suffer also from blindness and pain.

The portal for rare diseases and orphan drugs (Orphanet) indicates the incidence of LSCD with 1–5 over 10,000. In contrast, the incidence of LSCD with 3–4 over 1,000,000 in UK is even lower [1].

The disease can be divided into three groups: hereditary, acquired and idiopathic. The most common type is the acquired one, mostly due to chemical burn or infection. A further distinction is also made between partial and complete LSCD. For partial LSCD, there is locally limited destruction of the limbal epithelial stem cells. This leads to a vascularized pannus growing from this damaged area onto the corneal surface, which can considerably reduce vision. Complete LSCD, in which most of the limbal epithelial stem cells are destroyed, results in complete conjunctivalization and vascularization of the corneal surface in form of a pannus. The damage to the limbal area leads to disturbances in the properties of the stem cells located there. The barrier function of the stem cell niche is lost, and the regeneration of the corneal epithelium by the limbal epithelial stem cells is no longer guaranteed [2, 3].

The therapy of LSCD is often frustrating. Multiple surgeries are often required, commonly due to relapse. This is the reason why we decided to create a new surgical method to reduce the risk of LSCD relapse, improve outcomes, and reduce the number of surgeries for patients in the future. We have called this new method ALT (allogeneic limbal transplantation).

The present study introduces the surgical technique and first year results.

## Methods

This study included 14 patients (6 females, 8 males). Six of the affected eyes were left eyes, and eight were right eyes. The mean age of patients was 69 years (29–90 years). Five of the patients developed LSCD due to chemical burn. Nine of the patients got LSCD because of an infection. Out of these nine patients, the most frequent cause of LSCD was herpetic infection (Tables 1 and 2). The median follow-up period (for all patients) was 12 months. All the patients suffered from low visual acuity, recurrent corneal erosions, and vascularization.

**Table 1:** Summary of patients with LSCD due to ocular burn

No.	Gender	Age	Preop. BCVA	LogMAR^a^ Preop. BCVA	Postop. BCVA^b^	LogMAR^a^ Postop. BCVA	Number of PALT grafts	Complications	Previous surgeries	VA-limiting findings	FUP [months]
1	F	67	Hm	3.0	20/60	0.54	5	Erosion, Cataracta complicata	1 × PK	Cataracta complicata	22
2	M	47	20/200	1.0	20/50	0.40	3	Cataracta complicata	4 × AMT		17
3	M	55	Hm	3.0	Hm	3.00	4	Seidel positive after explantation of suture	2 × PK 1 × AMT	OA	15
4	F	89	Lpr	4.00	20/80	0.6	3	None	multiple PK		17
5	M	76	20/80	0.6	20/50	0.4	2	Cataracta complicata	1 × AMT		27

*FUP* follow-up period, *f* female, *m* male, *hm* hand motion, *lpr* regular light perception, *PK* penetrating keratoplasty, *AMT* amniotic membrane transplantation

^a^Representation of the BCVA in LogMAR according to Händel and Küchle, BCVA of lpr is defined as a LogMAR of 4.00

^b^Time of determining the postop. BCVA was the last follow-up time, OA: optic atrophy

**Table 2:** Summary of patients with LSCD due to infection

No.	Gender	Age	Preop. BCVA	LogMAR^a^ Preop. BCVA	Postop. BCVA^b^	LogMAR^a^ Postop. BCVA	ALT number	Complications	Previous surgeries	pathogen	VA-limiting findings	FUP [months]
1	M	87	Hm	3.00	hm	3.00	2	–	2 × PK	Herpes	Glaucomatous OA	17
2	M	60	Hm	3.00	20/240	1.10	4	vascularization	1 × PK	Herpes, chlamydia		16
3	M	78	Hm	3.00	20/200	1.00	5	–	1 × PK	Ulcer	OA, nonexud. AMD	17
4	F	76	Hm	3.00	20/600	1.60	1	–	2 × PK	Ulcer		1
5	F	70	Lpr	4.00	Lpf	4.00	2	–	ppV	Ulcer	OA	3
6	F	29	Hm	3.00	20/200	1.0	5	loose suture	1 × PK, 5 × ppV	Herpes	OA, PVR-re Ablatio retinae	4
7	M	80	Hm	3.00	20/360	1.2	6	–	3 × PK	Herpes		6
8	F	90	Fc	2.00	20/600	1.60	8	–	7 × PK	Ulcer		10
9	M	70	Hm	4.00	20/800	1.60	3	–	1 × PK	Herpes		1

*FUP* follow-up period, *f* female, *m* male, *hm* hand motion, *lpr* regular light perception, *fc* finger counting, *ppV* pars plana vitrectomy, *PK* penetrating keratoplasty

^a^Representation of the BCVA in LogMAR according to Händel and Küchle, BCVA of lpr is defined as a LogMAR of 4.00

^b^Time of determining the postop. BCVA was the last follow-up time, OA: optic atrophy

Diagnosis of LSCD was based on slit lamp demonstration (Table 3).

**Table 3:** Diagnosis of LSCD based on slit lamp demonstration

No.	Delayed corneal staining	Superficial new vessels	Scar tissue	Conjunctivalization	Whorld epithelium	Neurotrophic component	Loss of limbal palisades of Vogt
1	Yes	Circular	Circular	Circular	Yes	Yes	Yes
2	Yes	Circular	Circular	None	Yes	Yes	Yes
3	Yes	Circular	8 ch	4 ch	Yes	Yes	Yes
4	Yes	Circular	Circular	7 ch	Yes	No	Yes
5	Yes	7 ch	6 ch	2 ch	Yes	Yes	Partially
6	Yes	Circular	Circular	10 ch	Yes	Yes	Yes
7	No	Circular	Circular	9 ch	No	Yes	Yes
8	Yes	11 ch	10 ch	6 ch	Yes	Yes	Yes
9	Yes	6 ch	None	None	Yes	Yes	Partially
10	Yes	Circular	8 ch	4 ch	Yes	Yes	Yes
11	Yes	Circular	Circular	5 ch	Yes	Yes	Yes
12	Yes	Circular	6 ch	5 ch	Yes	Yes	Partially
13	Yes	10 ch	Circular	None	Yes	Yes	Yes
14	Yes	10 ch	8 ch	4 ch	Yes	Yes	Partially

*ch* clock hour

### Preoperative setting

Each of the patients underwent a best-corrected visual acuity (BCVA) test, complete ophthalmologic examination with a slit lamp, fundoscopy or ultrasound examination, photodocumentation, optical coherence tomography of the anterior segment (AS-OCT), and, if possible, videokeratography.

### Surgical procedure

**Fig. 1:**
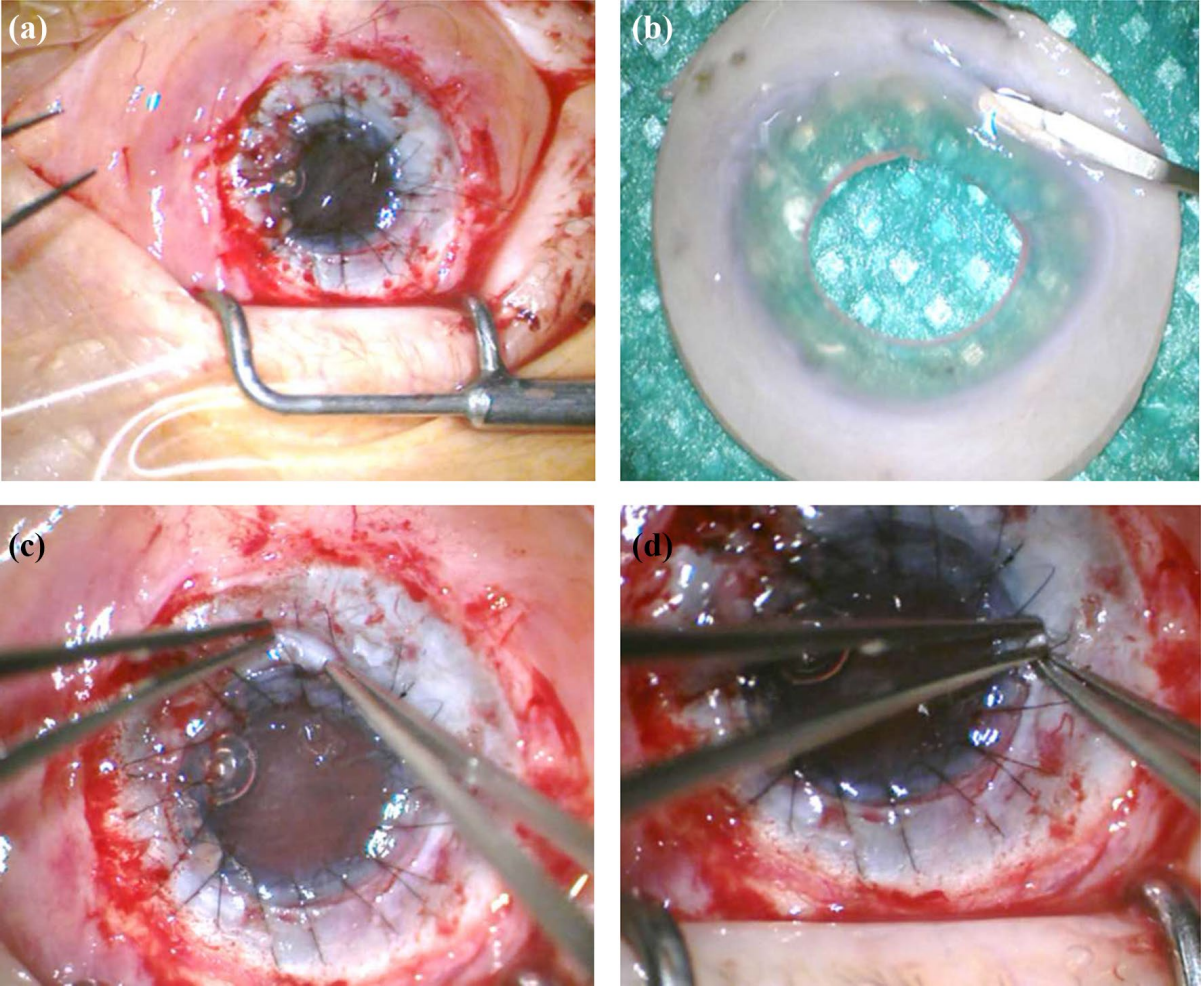
Intraoperative pictures of ALT surgery. **a** Finding after PK was performed. **b** Preparation of ALT pieces. **c**, **d** Placing the ALT pieces under the sutures

**Fig. 2:**
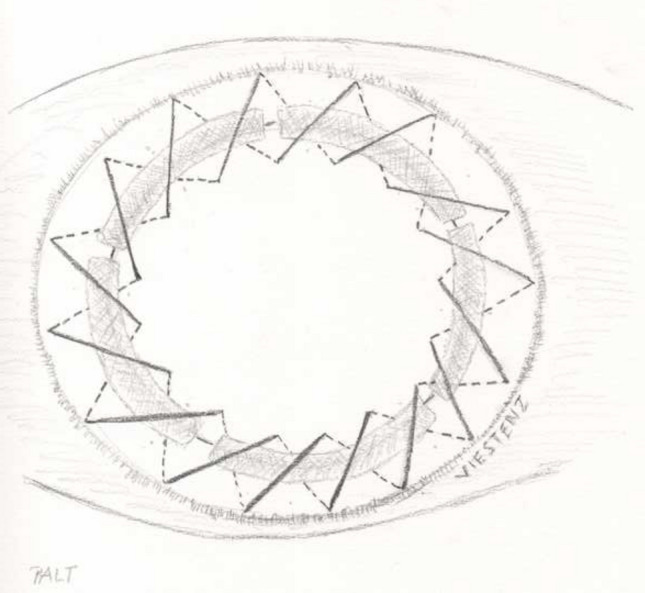
Principle of ALT combined with perforating keratoplasty. The grayish semilunar pieces above the donor–recipient interface are the ALT pieces, fixed using double-running 10–0 nylon sutures

ALT was carried out under general anesthesia by two surgeons (AV, AV). First, disinfection of skin and conjunctiva with iodine was performed to create sterile conditions. The superior and inferior muscles were fixated. Then a complete excision of the corneal pannus was necessary. When the whole pannus was removed, we trepanned the graft from the donor cornea using a Hessburg-Barron trephine system. Afterward, we performed full-thickness corneal trephination in the patient’s eye. In some cases, single sutures were used, whereas in other cases double running sutures were used in combination with single sutures (Fig. 1a). After suturing, preparation of the ALT started. One to eight limbal parts out of the sclerocorneal donor tissue were prepared, to avoid exerting too much pressure on the limbus and prevent the stem cells from getting damaged. The amount of ALT pieces used was adapted according to the chosen diameter of the corneal graft and the expansion of the underlying LSCD. In complete LSCD, we used as much pieces as we needed for the whole circumference. In partial LSCD, we limited the number of pieces to the involved area. In addition, the number of pieces was dependent of their size. These tissue parts were placed under the sutures. Only the sutures kept the ALT pieces in place, covering the donor and recipient tissues (Figs. 1b–d and 2). The ALT pieces were set at the positions where the strongest vascularization in the corneal tissue had been identified. Finally, a contact lens was placed to protect the small ALT pieces from getting lost due to mechanical stress caused by blinking. In two cases, the keratoplasty was combined with an open-sky cataract surgery. In some cases of massive vascularization of the cornea, bevacizumab was injected subconjunctivally. We performed amnion membrane patch transplantation additionally in one case. As the patch is loosening in the first two to four weeks in average with possible alteration of the ALT-pieces, we decided rather to keep the contact lens longer than to do amniotic membrane transplantation regularly in the other cases.

The contact lens covered the corneal transplant with the ALT pieces at least for four weeks.

Intraoperative management included intravenous carboanhydrase inhibitor (500 mg) and prednisolone (200 mg). For postoperative therapy, patients received topical unpreserved dexamethasone (tapering off over 6 to 8 months, one drop per day remaining), unpreserved ofloxacin (until corneal epithelial closure), and tear substitutes as well as systemic prednisolone over at least three weeks. Dependent on general condition of the patients, immunosuppressive therapy with mycophenolate was added for the whole follow-up period. Two patients received topical cyclosporine 1 mg/ml. In cases with underlying herpetic infection, we treated topical with ganciclovir and systemic treatment with aciclovir (400 mg 5 times per day for 6 weeks followed by 400 mg twice per day for one year).

We did follow-up examinations every two to three months, including a BCVA test, ophthalmologic examination with a slit lamp, fundoscopy, photodocumentation, optical coherence tomography of the anterior segment, and videokeratography.

## Results

The median average of the visual acuity (LogMAR) preoperative was 2.83, and it improved postoperatively to 1.50 with a significance of 0,001 (Table 4).

**Table 4:** Summary of the results and their significance

	All patients	Trauma group	Infectious group
Average VA preop* in LogMAR	2.83	2.32	3.11
Average VA postop* in LogMAR	1.50	0.99	1.79
Significance/*p*-value	0.01	0.12	0.003

*VA* visual acuity

For better comparability, the patients were organized into two groups based on the genesis of the LSCD: chemical burn and postinfectious group.

### Chemical burn

All five patients in the chemical burn group had undergone previous surgeries, three of them keratoplasty, and the others amniotic membrane transplantation.

Overall, none of the patients showed signs of relapse of LSCD during the postoperative follow-up period. No corneal neovascularization occurred in any patient. The BCVA was stable postoperatively in all patients compared to the preoperative BCVA. In four of five patients, visual acuity increased postoperatively (Table 1). The most common complication was the development of a cataract. One patient exhibited a positive seidel phenomenon after the sutures were explanted. None of the patients reported complaints at the follow-up examinations. The median follow-up period was 19.6 months. The median average in LogMAR of the preoperative visual acuity was 2.32; it increased postoperatively to 0.99 with a significance of 0.12 (Table 4). This shows a clear trend of better postoperative VA even if it is not significant.

**Fig. 3:**
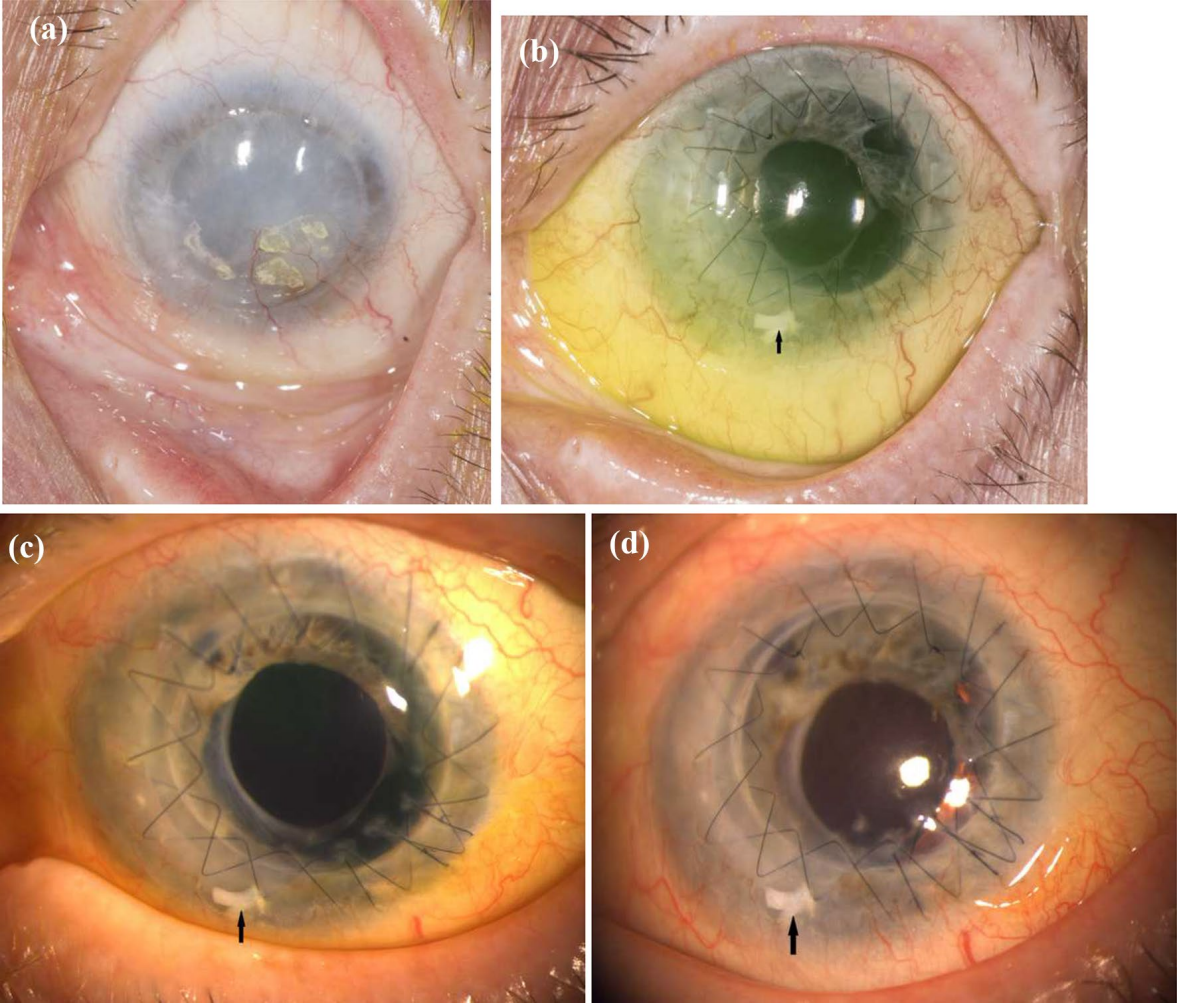
Clinical pictures of case 1 with LSCD caused by ocular burn. **a** Preoperative picture. **b** 2 months postoperative. **c** 12 months postoperative. (d) 18 months postoperative. Arrow: ALT piece

We will now give a detailed report on two of the five patients who suffered LSCD due to chemical burn:

*Case 1* was an 89-year-old woman who had suffered from LSCD for 50 years due to ocular burn caused by lye. She already had undergone keratoplasty thrice, the last one eleven years ago. Her BCVA before ALT surgery was light perception. The BCVA increased to 20/200 after two months postoperatively, up to 20/80 after 12 months. There were no complications, and the ALT grafts remained in their position during the whole follow-up period (Fig. 3).

**Fig. 4:**
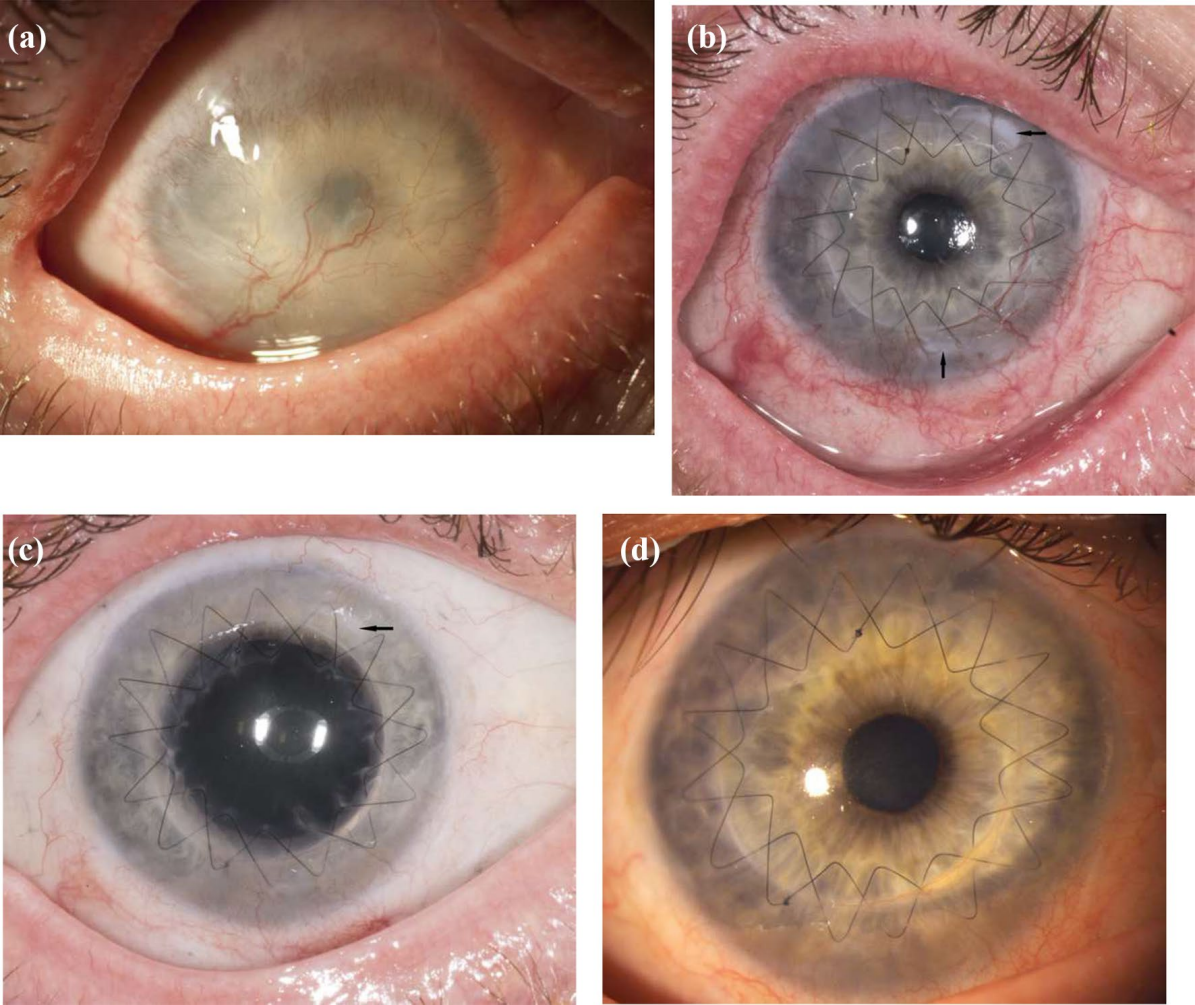
Clinical pictures of case 2 with LSCD caused by ocular burn. **a** Preoperative picture. **b** 1 month postoperative. **c** 4 months postoperative. **d** 21 months postoperative. Arrows: ALT pieces positioned under the sutures

*Case 2* was a 47-year-old man who got LSCD due to a work accident that resulted in ocular burn. Before ALT surgery was performed, he had undergone amnion membrane transplantation (AMT) on four occasions. His preoperative BCVA was 20/200, which increased to 20/50 after 14 months. The eye showed a mild complicated cataract, which already existed before ALT surgery (Fig. 4).

### Postinfectious group

Five of the nine patients in this group developed LSCD because of herpetic infection, one of them in combination with a chlamydia infection. The other cases developed LSCD in combination with corneal ulceration, but the infectious trigger remained unclear. Eight of the patients had undergone previous keratoplasty, and four of them more than once.

Only one of the nine patients showed new corneal vascularization in combination with a pannus. The other patients showed no new vascularization. The BCVA was stable postoperatively in two patients compared to the preoperative BCVA and increased postoperatively in seven patients (Table 2). The median follow-up period was 8 months. None of the patients made complaints during the FUP (follow-up) examinations. One patient had a loose suture, and other complications were not observed. The median average of the preoperative visual acuity (in LogMAR) was 3.11, which increased postoperatively to 1.79 with a high significance of 0.003 (Table 4).

We now present more details about two patients that suffered LSCD from infection.

**Fig. 5:**
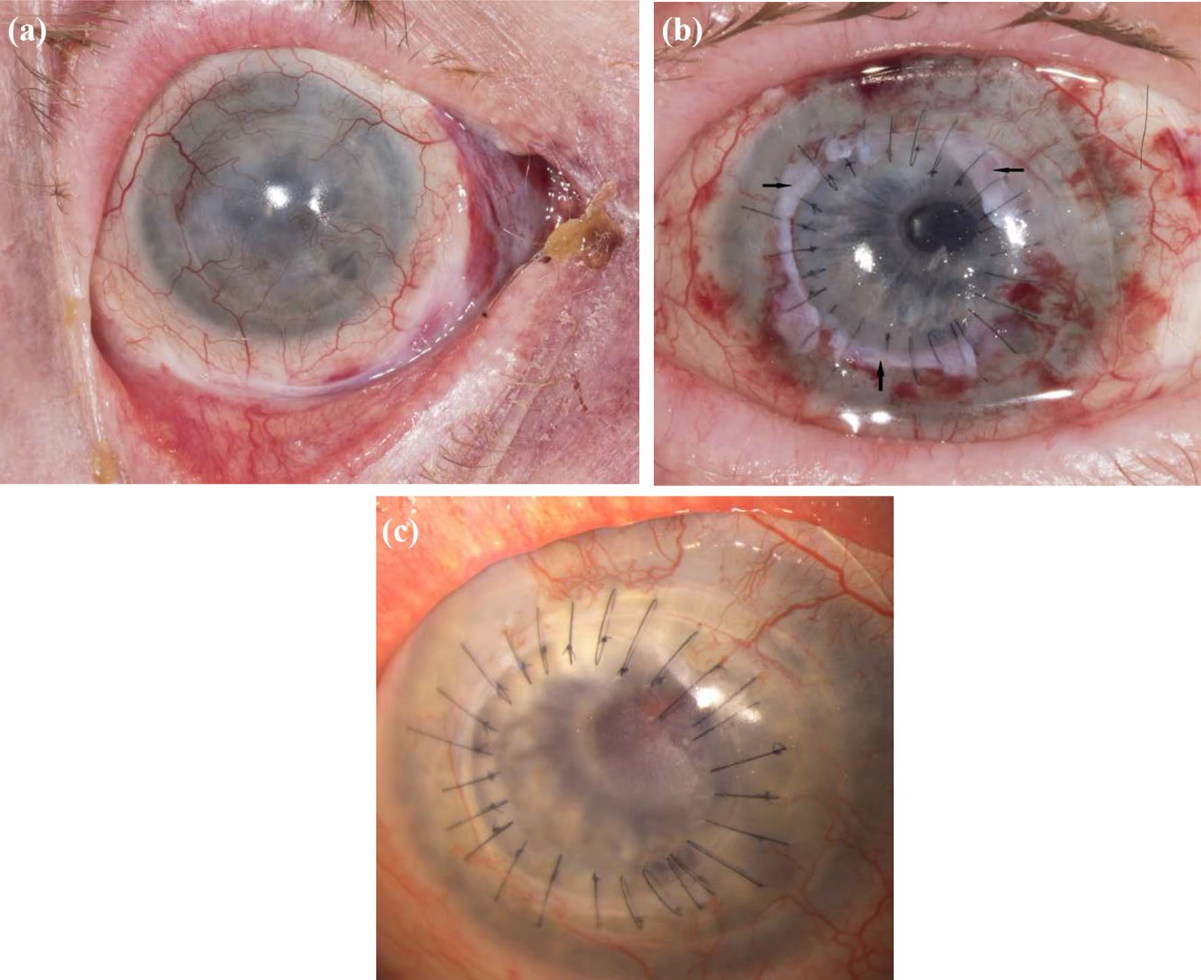
Clinical pictures of case 3 with LSCD caused by herpetic infection. **a** Preoperative picture. **b** 7 days postoperative. **c** 5 months postoperative. Arrows: ALT pieces placed under the sutures

*Case 3* was a 90-year-old woman who had already undergone PK on seven occasions. The last PK was performed twelve years ago. The cornea showed 12 clock hours of corneal neovascularization. Furthermore, she suffered from recurrent corneal ulcers. The preoperative BCVA was “finger counting”, and six months after the ALT procedure it had increased to 20/600. The ALT remained continuously in their position and no further corneal vascularization occurred after ALT surgery (Fig. 5).

**Fig. 6:**
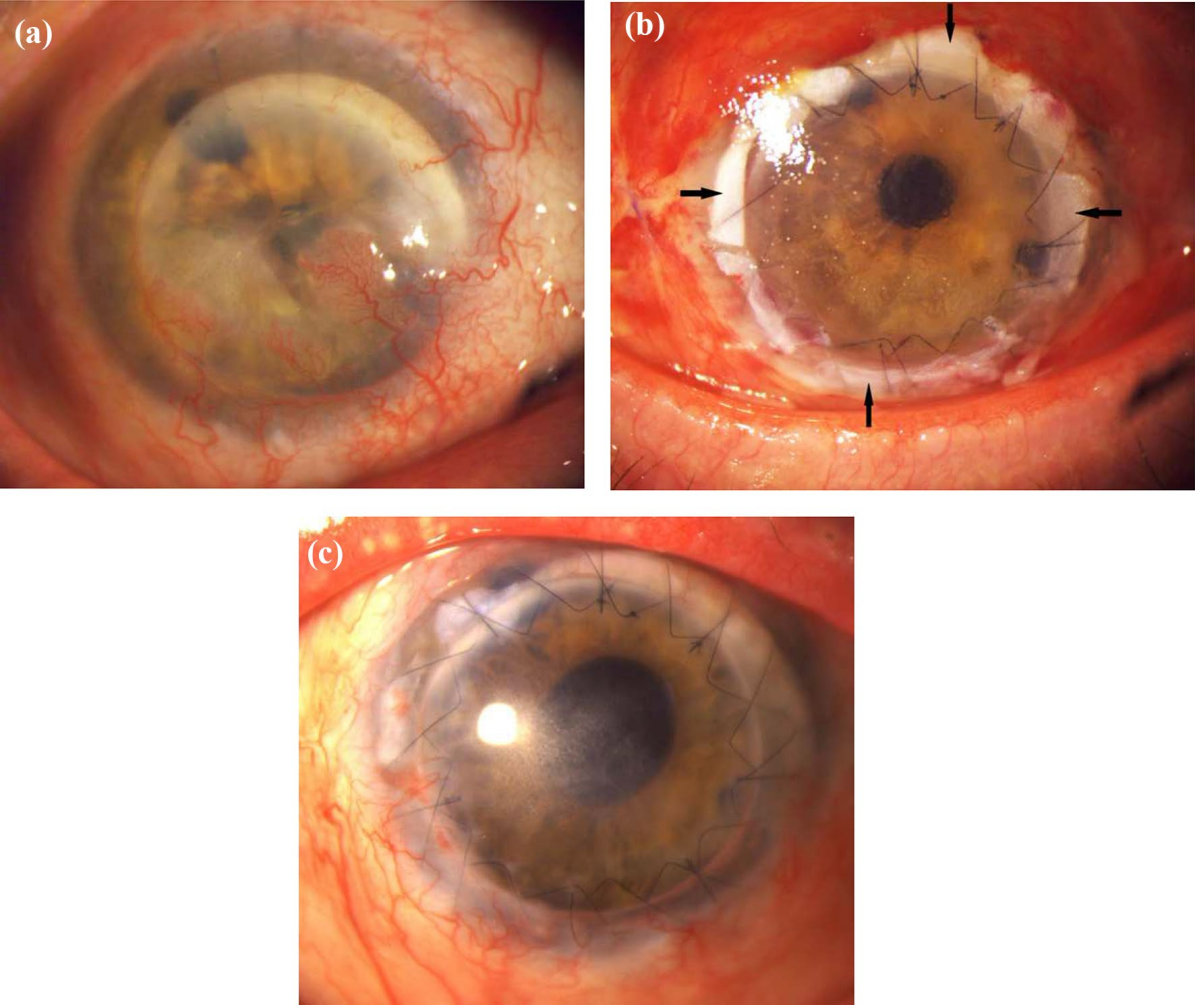
Clinical pictures of a patient with LSCD caused by herpetic infection. **a** Preoperative picture. **b** 3 days postoperative. **c** 3 months postoperative. Arrows: ALT pieces

*Case 4* (Fig. 6) as an 80-year-old man who suffered from recurrent herpetic infection which already required three PK. The preoperative BCVA was “hand motion”, and three months after ALT with PK it had increased to 20/360. No complications were observed in the FUP period of six months.

### Optical coherence tomography of the anterior segment

**Fig. 7:**
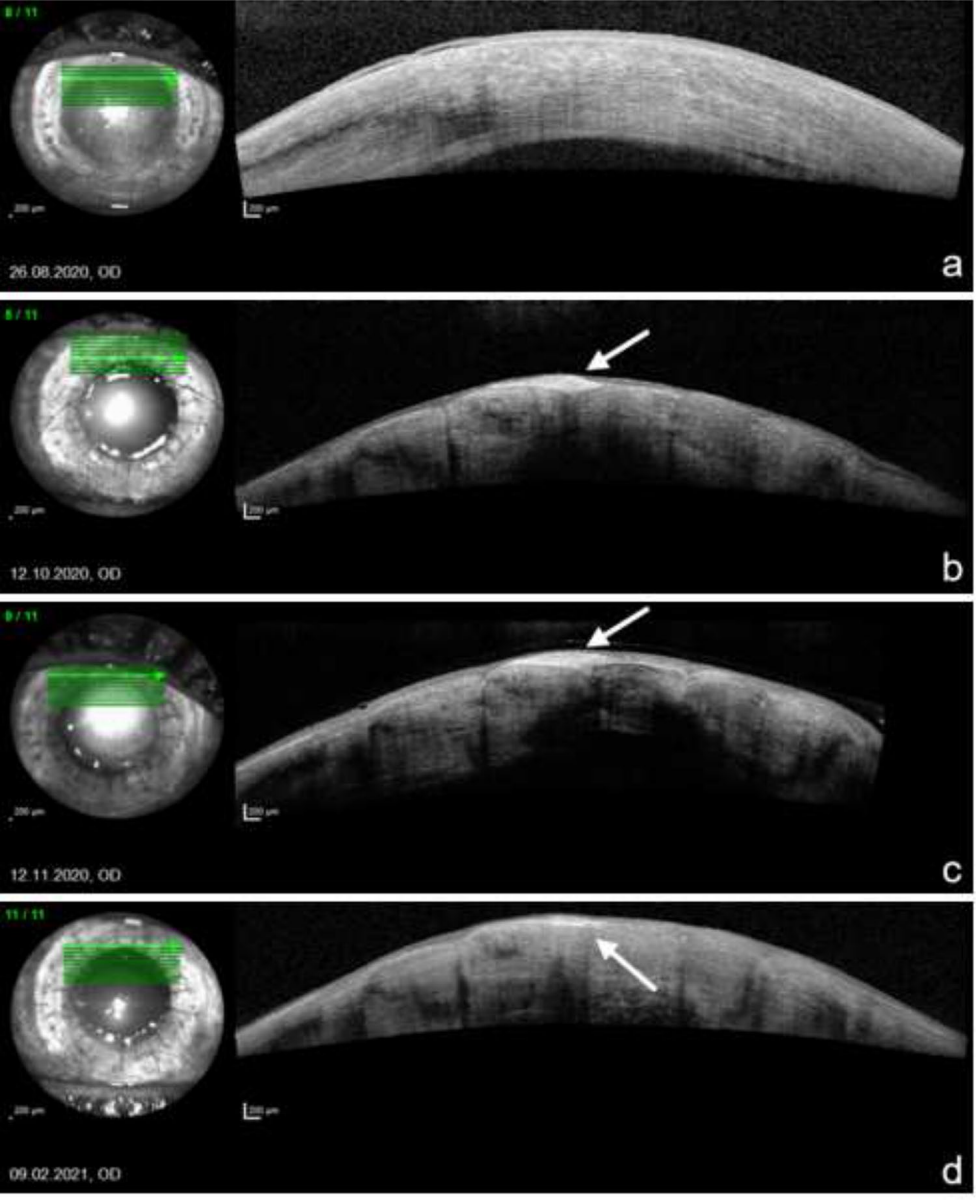
AS-OCT of the ALT-pieces during follow-up. **a** Preoperative thickened cornea with corneal pannus. **b** Six weeks postoperative, subepithelial ALT-piece (arrow) fixed between the sutures. **c** Ten weeks postoperative. **d** Five months postoperative, ALT-piece becomes thinner while epithelial layer is thickening

We could observe the surveillance of the ALT-pieces in optical coherence tomography of the anterior segment over the whole follow-up time. The pieces become thinner and more transparent but remain subepithelial in place (Fig. 7).

## Discussion

There are several therapeutic options for treating LSCD. Restoring of the corneal epithelium depends on the presence of remaining limbal epithelial stem cells (LESC). In cases with no remaining stem cell reserves, the cornea must be reseeded with new LECS.

In recent years, the possibility of ex vivo cultivation of limbal stem cells and subsequent transplantation has been established, which is known as cultivated limbal epithelial transplantation (CLET). This process was first described in 1997 [4]. In CLET, small limbal biopsy (2 × 2 mm) is performed either allogenously or autologously. This way, the limbal epithelial stem cells are cultivated and expanded on human cryopreserved amniotic membrane or on a fibrin-based substrate. Two different cultivation techniques have so far been developed. In the first technique, the explant technique, the removed cells are cultured in vitro on human amniotic membrane for 2–3 weeks. After this, the cells are re-transplanted onto the diseased eye [5]. In the second technique, the suspension method, the limbal stem cells are enzymatically isolated from the tissue. These cells are cultured on fibrin substrate carriers or on an amniotic membrane. After confluence, cells are retransplanted onto the diseased eye [4]. The CLET procedure is particularly suitable for LSCD caused by burns (including chemical burns), which are the most common cause of the disease [6]. In 2015, the CLET technique was approved for use in the manufacture of a stem cell-based drug in the European Union named Holoclar. The drug contains autologous human corneal epithelial cells with about 3.5% limbal stem cells on a fibrin membrane and is only approved for the therapy of unilateral and bilateral LSCD caused by burns or chemical burns. This therapy requires an intact limbal area of 1–2 mm^2^ from which a biopsy is taken, and the cells obtained in this way are expanded ex vivo. The cells are expanded until the optimal cell count of 79,000 to 315,000 cells per cm^2^ is reached. The membrane with the expanded cells is applied to the defect area. This method minimizes the risk of transplant rejection and iatrogenic LSCD. In a multicenter, uncontrolled, retrospective case series cohort study with 106 patients, a positive result was found in 72.1% of the cases. People's visual acuity has been improved, and the number of people with symptoms has been decreased [7].

In 2012, Sangwan et al. developed a procedure called simple limbal epithelial transplantation (SLET) [8]. In this surgical technique, a 2 × 2-mm tissue sample is taken from the limbal region of a healthy eye and divided into many small pieces. These pieces are applied by fibrin glue onto a fresh amniotic membrane, which is attached to the diseased eye in advance. The small pieces are expanded in vivo. The ideal patient for this procedure has unilateral LSCD. Postoperatively, a contact lens is placed onto the eye to protect the transplanted grafts and antibiotic and steroid-containing eye drops are used. Proliferation of the corneal epithelium starting from the transplants becomes visible after the second postoperative day and epithelialization of the cornea is complete after 4–12 days [9]. A 2020 review summarized 404 cases of SLET. A success rate of 83% (stable corneal epithelium and absence of vascularization) was obtained as well as an improvement in visual acuity in 69% of cases. The most common postoperative complications were focal recurrence of LSCD, progressive conjunctivalization, progressive symblepharon, and keratitis. Also, a risk of iatrogenic LSCD was described [10].

All these described methods require one eye with a healthy limbal region or at least an undamaged part of the limbal region to obtain a sample of the limbal stem cells. So, these methods are not suitable for patients with both-sided LSCD. Patients often refuse a biopsy of the healthy eye. One important advantage of the ALT procedure is that no separate donor eye for the transplantation of the limbal region is necessary. The transplantation is allogeneic, and the limbal stem cells are residual tissue of the keratoplasty from the same donor. So ALT can be carried out in all patients with LSCD. Even patients with a totally damaged limbal region in both eyes can be included in this procedure. Because of this, there is no risk of iatrogenic LSCD. Due to the iatrogenic damage of the limbus during the biopsy when SLET or CLET is performed, there is always a risk of iatrogenic LSCD in the biopted eye.

Another advantage of ALT is that there are no special requirements necessary for cultivation. To perform CLET, a specialized laboratory and corresponding laboratory staff are crucial. Also, the Holoclar procedure, which is quite similar to CLET, needs a specialized team for culturing and transportation of the obtained limbal stem cells.

In contrast to the already mentioned procedures, ALT includes keratoplasty. Of course perforating keratoplasty is more invasive than a biopsy or stem cell transplantation alone. Nevertheless, patients who undergo CLET/Holoclar® or SLET often need more surgeries than a surgery for implantation of limbal tissue. Therefore, additional keratoplasty is also often necessary after the biopsy has been performed to get increased BCVA. The reason for is to remove the stromal-located corneal scars or reduce clouding, which often remain even if there are limbal stem cells again. So, to get increased BCVA, perforating keratoplasty is performed.

Some other surgical techniques also include keratoplasty. One method is the penetrating limbo-keratoplasty. Here a graft with a diameter of 7.7–10.0 mm is transplanted with a proportion of 40% limbal tissue at the graft´s circumference [11, 12]. The long-term result over five years showed that 14% of the untyped grafts remained clear. This shows that there is probably a higher risk of rejection, because a high immunogenic tissue is transplanted. To reduce this risk, higher-dosed immunosuppression is perhaps necessary. The outcome is much better when HLA-typed transplants are used. Here, 41–65% of the grafts are transparent after five years depending on the number of mismatches of the HLA status [13]. This leads to the finding that the immunogenic reaction when using a HLA-typed graft is lower. The disadvantage of course is that the waiting period for a suitable transplant is much longer than for an untyped graft.

Viestenz et al. reported on a patient with bilateral corneal burn and corneal perforation. They placed a 15-mm corneoscleral graft over the anterior segment without removing the central cornea. After 2–3 weeks, a 23-gauge vitrectomy was performed to remove the collagenolytic central recipient cornea. The corneal graft remained transparent for 3–5 years. The allogeneic stem cells were adjacent to the cornea [14] and possibly created a new limbal region. Nevertheless, rejection happened after 3–5 years.

These two surgical techniques show that the limbal region is highly immunogenic and rejection often cannot be avoided. When using the ALT technique, the explanted transplants are placed not in the limbal region but further to the center of the cornea. This distance to the immunogenic limbal region of the patients may reduce the risk of rejection. Of course, there are no long-term results for the ALT technique in terms of the rejection rate, but the first results have so far shown no rejection.

Nevertheless, immunosuppression is necessary because allogenic tissue is used for keratoplasty and ALT. For this, prednisolone was used as well as topic and systemic therapy. Prednisolone has proved effective in preventing rejection [15], although there are well-known side effects of immunosuppression with corticosteroids like development of cataract, glaucoma, arterial hypertension, increased blood sugar, weight gain, mental abnormalities, and much more. So, the indication for long-term therapy with corticosteroids has to be evaluated carefully.

Another surgical method that includes PK for LSCD treatment is the so-called SCET (simple conjunctival epithelial transplantation), which was developed in 2020 by Sakimoto et al. They suggest that transplantation of autologous conjunctival cells is a promising therapeutic procedure in patients with LSCD. The idea of this procedure is based on the SLET technique. To perform a SCET, the pannus is removed and, afterward, PK or lamellar keratoplasty is carried out. Then AMT is placed over the ocular surface. Once a 4 × 3-mm piece of the temporal superior bulbar conjunctiva has been separated, 10–15 pieces of this conjunctival tissue are fixed with fibrin glue on the amniotic membrane. The amniotic membrane is then covered with a contact lens. Four patients with LSCD originating from different causes underwent this surgical procedure and showed increased visual acuity and stable ocular surfaces [16].

In contrast to this procedure, ALT uses allogenic limbal tissue. It is likely that ALT pieces also contain conjunctival tissue because of the localization of the limbus. In the SCET procedure, conjunctival cells can accept corneal epithelial properties, resulting in a transparent cornea and a stable ocular surface. This has already been shown in two other publications [17, 18]. It leads to the assumption that the conjunctival part of the ALT also has an impact on the transparency and stability of the transplanted corneal tissue. However, the mechanism behind how the conjunctival tissue suddenly changes to the corneal epithelial cells is still unknown.

Another surgical method whereby the conjunctival tissue is transplanted is the conjunctival limbal autografting (CLAU), which was developed in 1989. Here, two conjunctival-limbal autotransplants (120 degrees of the corneal circumference) are transferred to the affected eye [19]. The improvement in visual acuity is 25–100% [20, 21]. This leads to the assumption that the transplanted conjunctival tissue gives a positive effect to the outcome. A disadvantage of this method is, as already mentioned above, a high risk of iatrogenic LSCD because a quite large part of the limbus of the unaffected eye is explanted.

Patients who undergo ALT require just one surgery if no postoperative complications develop. The exact functionality of how the ALT pieces integrate into the tissue and how the limbal stem cells from the transplant reach the limbus has not yet been clarified. Our study shows that patients who underwent ALT surgery seem to create a new intact limbal region. This is indicated by the lack of corneal vascularization and the intact postoperative epithelium. It is not clear whether the limbal stem cells migrate from the ALT transplant in the direction of the damaged limbus and implant there or whether a new limbal region is created in the area of the ALT. This study only includes 14 patients, and the median follow-up period was 12 months. Therefore, further investigations are necessary to prove the long-term results of PK combined with ALT.

The present study introduces the surgical technique and first year results without disclosure of patients for any reason. Feasibility of the surgery and surveillance of the transplanted tissue as well as integration of the ALT-tissue were our major points of interest of this pilot study. Medium- and long-term follow-up will be reported.

## Conclusions

The ALT plus PK procedure seems to be a promising surgical technique for the treatment of patients with LSCD. No risk of iatrogenic LSCD was noted in the study sample. ALT can easily be carried out during perforating keratoplasty, so no separate donor tissue is necessary. The exact mechanism of how ALT works remains unclear.

## Data Availability

The datasets generated during and/or analyzed during the current study are available from the author on reasonable request.
